# Reactions and countermeasures of medical oncologists towards the incoming COVID-19 pandemic: a WhatsApp messenger-based report from the Italian College of Chief Medical Oncologists

**DOI:** 10.3332/ecancer.2020.1046

**Published:** 2020-05-15

**Authors:** Livio Blasi, Roberto Bordonaro, Nicolò Borsellino, Alfredo Butera, Michele Caruso, Stefano Cordio, Di Cristina Liborio, Francesco Ferraù, Dario Giuffrida, Hector Soto Parra, Massimiliano Spada, Paolo Tralongo, Roberto Valenza, Francesco Verderame, Stefano Vitello, Filippo Zerilli, Dario Piazza, Alberto Firenze, Vittorio Gebbia

**Affiliations:** 1 Medical Oncology Unit, Arnas, Ospedale Civico, Palermo, 90100, and National President of CIPOMO, Italy; 2 Medical Oncology Unit, Arnas, Ospedale Garibaldi, Catania, 95100, Italy; 3 Medical Oncology Unit, Ospedale Buccheri La Ferla, Palermo, 90100, Italy; 4 Medical Oncology Unit, Ospedale San Giovanni di Dio, Agrigento, 92100, Italy; 5 Medical Oncology Unit, Istituto Clinico Humanitas, Catania, 95100, Italy; 6 Medical Oncology Unit, Ospedale Paterno Arezzo, Ragusa, 97100, Italy; 7 Medical Oncology Unit, Ospedale Vittorio Emanuele, Castelvetrano, 91022, Italy; 8 Medical Oncology Unit, Ospedale San Vincenzo, Taormina, 98039, Italy; 9 Medical Oncology Unit, Istituto Oncologico Mediterraneo, Viagrande, 95029, Italy; 10 Medical Oncology Unit, Policlinico, Catania, 95100, Italy; 11 Medical Oncology Unit, Ospedale Giglio, Cefalù, 90015, Italy; 12 Medical Oncology Unit, Ospedale Umberto I, Siracusa, 96100, Italy; 13 Medical Oncology Unit, Ospedale Vittorio Emanuele, Gela, 93012, Italy; 14 Medical Oncology Unit, Ospedale Cervello-Villa Sofia, Palermo, 90100, Italy; 15 Medical Oncology Unit, Ospedale Sant’Elia, Caltanissetta, 93100, Italy; 16 Medical Oncology Unit, Ospedale San Antonio Abate, Trapani, 91100, Italy; 17 GSTU Foundation, Palermo, 90100, Italy; 18 Risk Management Unit, Policlinico, Palermo, 90100, Italy; 19 Medical Oncology Unit, Ospedale La Maddalena, University of Palermo, Palermo, 90100, Italy

**Keywords:** WhatsApp messenger, medical oncologists, reactions, action taken, COVID-19 outbreak, sentimental analysis

## Abstract

**Background:**

This descriptive, unplanned investigation has been undertaken to report reactions, attitudes and countermeasures which have been put in place and implemented by medical oncology units facing the COVID-19 outbreak in Southern Italy.

**Materials and methods:**

Data have been retrospectively obtained from the time-related analysis of conversations via a WhatsApp messenger-based group chat between the medical directors belonging to the Italian College of Medical Oncology Directors. Overall number, intensity and time trend of conversations related to reactions during the 4 weeks of observation related to the crucial events which occurred between 24 February and 28 March, 2020 2020 are included. A sentiment analysis of conversations was also carried out.

**Results:**

We report 956 conversations among 19 medical oncology units related to reactions to the crucial events, such as epidemic spread, Government ordinances and guidelines during the 4 weeks of observation. Data show significant awareness of problems linked to the COVID-19 spread among oncologists and rapid diffusion of countermeasures. Actions taken were correlated time wise to crucial events. A correlation between conversations and the volume of activity of oncology units was found. By analysing the sentiment analysis of raw data, positive emotions were reduced in percentage over the weeks. A significant increase in negative emotions was observed as the outbreak impacted on the healthcare system.

**Conclusion:**

In our experience, the WhatsApp instant-messaging system seems to be a useful tool to share news and reactions between medical oncologists to rapidly implement necessary health measures and answers to most cancer patients’ needs and queries in the COVID-19 pandemic scenario.

## Background

In the last couple of months, the new coronavirus COVID-19 has spread very rapidly in Northern Italy causing a severe overload for the National Health System due to a sharp increase of new patients with severe respiratory distress needing prompt hospitalisations in pulmonary and intensive care units which were numerically unprepared for the sudden increase in new daily requests [1–3]. As very recently reported, the rapid diffusion of the epidemic may be attributed mainly to undocumented infections estimated to be as high as 86% before travel restrictions [4].

Data from the COVID-19 epidemic in China reported a significant mortality rate with the warning that aged patients and those with concomitant underlying disease are particularly at risk of death [5, 6]. Among these patients, those affected by cancer, in particular lung carcinoma, and receiving immune-suppressive therapies represented a significant percentage of the whole series [7–10].

The COVID-19 infection is spreading southbound in Italy, and in Sicily, the number of new cases per day is rapidly growing [2]. Therefore, the oncology community and administrative or government institutions reacted, preparing to face a possible wide spread as in Northern Italy. In this paper, we report the reactions, the attitudes and countermeasures which have been put in place and possibly implemented by most medical oncology units. We employed data obtained from the time-related analysis of conversations via a pre-existing WhatsApp messenger-based group chat between the medical directors belonging to the Italian College of Medical Oncology Chiefs (CIPOMO—Collegio Italiano dei Primari Oncologi Medici Ospedalieri. www.cipomo.it).

The WhatsApp Instant-Messaging system (WM) is a useful and easy tool to rapidly share any information useful to participants in various clinical settings [11–13]. Its diffusion and usage among healthcare professionals is mainly due to the real experience of many advantages and benefits reported in clinical practice, which can be summarised in a greater communication flexibility and a more streamlined management of some workflows [14].

Sentiment analysis is a natural language processing technique employing computer algorithms to extract subjective information from written text and help in identifying the strength of the positive and negative tone of the message [15, 16]. Recently, this new technique has been used to investigate, analyse and predict people's behaviour in the healthcare field [17–20]. Sentiment analysis of healthcare settings could help investigators to understand better how people talk and feel about specific topics or conditions. However, analysing the mood is not easy because emotions are often mixed or ambivalent. Mixed emotions describe the simultaneous experience of different combinations of opposite emotions [21]. Since positive and negative emotions can occur more or less at the same time, the analysis of the emotions expressed in the texts may be challenging [21]. The use of sentiment analysis is particularly relevant in a social media context, as it has become a natural environment where people can share and search for health information. Social media content has, due to the type of information found and the emotional aspects expressed, an impact on individuals and their decision-making process [21]. Some sectors of the population have shown a change in health-related behaviour through the increasing search for www-based health information. Recent examples of this change include ‘spontaneous telemedicine’, where doctors have changed the behaviour of themselves and their colleagues through the use of Instant-Messaging, for example, WhatsApp [22].

Due to the COVID-19 outbreak in Italy, the conduction of surveys among health workers, even if administered online, is challenging because they require the full collaboration of the participants. The data obtained from the retrospective analysis of the conversations carried out in WhatsApp groups, similar to real Focus Groups, can represent a key online tool to understand this social problem in a non-invasive way.

This study aims to explore the impacts of the COVID-19 public health emergency and the impact it had on the organisation of hospital oncological activities in southern Italy, on the reactions of oncology unit directors during the crucial phases of the outbreak and eventually the impact on the health of cancer patients themselves.

## Materials and methods

### Data collection

On 27 November, 2017, CIPOMO set up a WhatsApp messenger-based group chat to allow rapid, non-official data exchange between most directors of medical oncology units in Sicily. The Sicilian regional section of the CIPOMO accounts for 14 directors of hospital oncology divisions/departments. Five other units not belonging to the CIPOMO were included in the WhatsApp messenger-based group for a total of 17 units actively participating. The main aim of this paper is to report descriptive time-related analysis of the reactions and attitudes of medical oncology units to the incoming spread of the COVID-19 infection in Southern Italy and, in particular, Sicily, which has a population of 5.027 million as last assessed in 2018. After previously written e-approval of all participants in respect to the Privacy Acts Law in force in Italy, we retrospectively analysed all conversations recorded in a WhatsApp messenger-based group starting from the first message on COVID-19 sent on 24 February until 28 March, 2020. An epidemiology expert (A.F.) and a project/data analyst manager (D.P.) evaluated the messages, categorised the data into five classes by topics homology and plotted against time and fast-evolving major government ordinances, scientific societies’ guidelines and infection prevalence data. Messages were transformed in data-sheets for analysis. Ethics approval was not required because the research survey was considered morally acceptable and could not risk harming the study participants or other subjects. Moreover, Italian legislation does not require ethics approval for research not involving patients.

### Sentiment analysis

Sentiment analysis is the computational study of people’s opinions, sentiments, emotions and attitudes, it is also known as opinion mining [23]. To better understand how oncologists talk and feel about this potential critical health topic, we also apply to the data-set a sentiment analysis using an unsupervised approach of Natural Language Processing. The lexicon-based approach implies calculating the orientation of a document from the semantic orientation of the words contained in the document. The text classification approach requires the construction of classifiers from labelled instances of text. The semantic orientation scores are then aggregated into a single score for the text. Messages have been classified by positive emotions (anticipation, trust and joy) and negative emotions (disgust, fear and sadness) according to Plutchik’s primary human emotions classification [24]. The analysis was performed using R: A Language and Environment for Statistical Computing version 3.6.3 [25] with the package Syuzhet: Extract Sentiment and Plot Arcs from Text [26]. The NRC Word-Emotion Association Lexicon [27], available via open access also in the Italian language, which contains 10,170 lexical items coded for Plutchik’s classification and implemented in the Syuzhet R package, associates an emotion (or more than one emotion) to each of the 10,170 lexical items. Given a word and emotion X, the NRC Word-Emotion Association Lexicon associates a score (range: 0 to 1) with it. A score of 1 indicates that the word conveys the highest amount of emotion X. A score of 0 indicates that the word conveys the lowest amount of emotion X.

### Data cleaning

Messages containing links to web pages, videos, audio and images were excluded. Emoji were converted into the respective words; the final sample was cleaned for analysis by using a process of basic normalisation, stop word removal, normalisation of user mentions, lemmatisation and nonprintable character removal.

### Statistical analysis

Descriptive statistics were used to show participants’ characteristics. Percentages are reported approximated to the nearest unit. Results were analysed, applying data to a contingency table and employing a chi-square test for trend. An exploratory analysis of subgroups was performed according to the following variables: age (<50, 50–59, ≥60 years) and years of oncology experience (<15, ≥15). All participants were male, so we did not analyse gender.

## Results

Conversations between the 19 participating medical oncology units were retrospectively recorded from 24 February, 2020, to 29 March, 2020, for a total of four consecutive weeks. Table 1 shows the characteristics of medical oncology directors participating and messages statistics. Comprehensive cancer centres were 26% of participating centres, general hospitals 63% and academic institutions 11%. A total of 956 messages were analysed and categorised into five classes of reaction by topic homology. Conversations were classified as a reaction to A) measures taken by the Italian Government; B) measures taken by local health government agencies; C) measures taken by the oncology scientific societies; D) to cases of contagion of patients in one’s facilities and E) to the occurrence of infection among medical oncologists.

**Figure 1.**
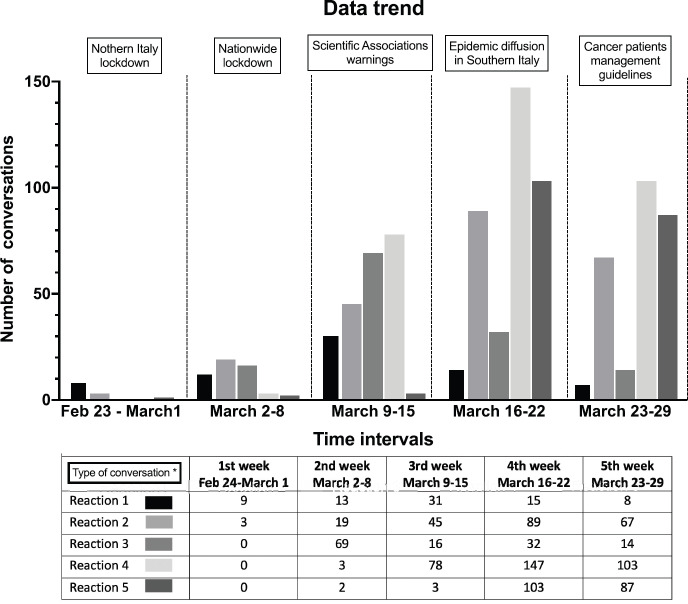
Weekly intensity in relation to the crucial events. (*) 1) measures taken by the Italian Government; 2) to measures taken by health government agencies; 3) to measures taken by the scientific oncology societies; 4) to cases of contagion of patients in one’ own facilities and 5) to the occurrence of infection among medical oncologists.

For each week under observation, the significant events that contributed to a profound change in the regular working routine and intense psychological pressure were identified (Figure 1). In the first week (February 23 to March 1), the Italian Government instituted a lockdown in the regions of Northern Italy where the outbreak overwhelmingly started, while in the following week, it extended to the entire nation due to epidemic diffusion [28]. In the third week (March 9–15), the Italian Society of Medical Oncology (Associazione Italiana di Oncologia Medica—AIOM—www.aiom.it), together with the CIPOMO and the Board of University Medical Oncologists (Collegio degli Oncologi Medici Universitari—COMU), issued a note containing the indications and behavioural norms for the management of cancer patients as related to the infection risk from COVID-19 [29] (Appendix 1). The fourth and fifth weeks saw a progressive increase in infection cases among the Sicilian population, the first infections among cancer patients and doctors, and the release of recommendations and guidelines for cancer patient management by the Ministries of Health and Internal Affairs [30].

**Figure 2.**
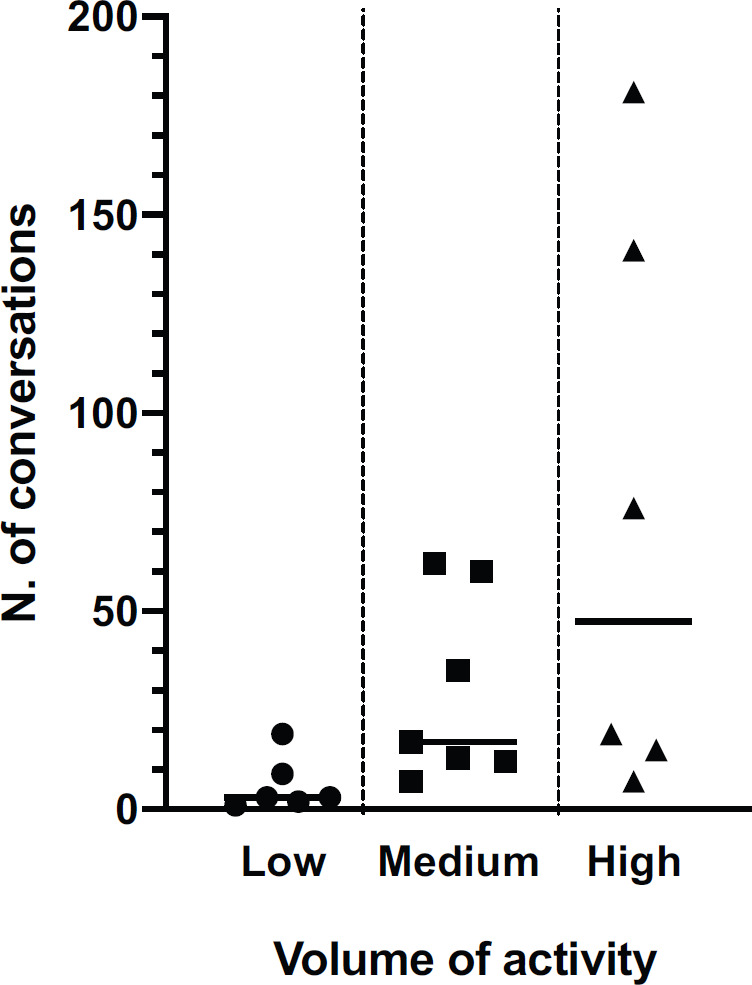
Correlation of the number of conversations and activity volume of participating centres.

Figure 1 shows the number and intensity of conversations related to reactions during the 4 weeks of observation related to the crucial events that occurred. The raw number of conversations abruptly increased from 12 in the first week to 106 in the second week and progressively increased to 173, 386 and 279 in the following 3 weeks. This trend was statistically significant (*p* < 0.0001, χ^2^ test for trend). Figure 1 also shows the time-dependent of different types of reactions. The conversation concerning possible cases of contagion of patients in one’s facilities (reaction D) and its consequences increased steadily from week 2^nd^ to 4^th^. Conversations related to measures suggested by the scientific oncology societies showed a spontaneous increase in the week before the publication of guidelines and a sharp peak immediately after the release of guidelines and suggestions. As shown in Figure 2, absolute numbers of messages were correlated to the importance of participating centres in terms of treatments delivered per month (high-volume versus medium and low-volume). No difference was observed for age and years of experience.

**Figure 3.**
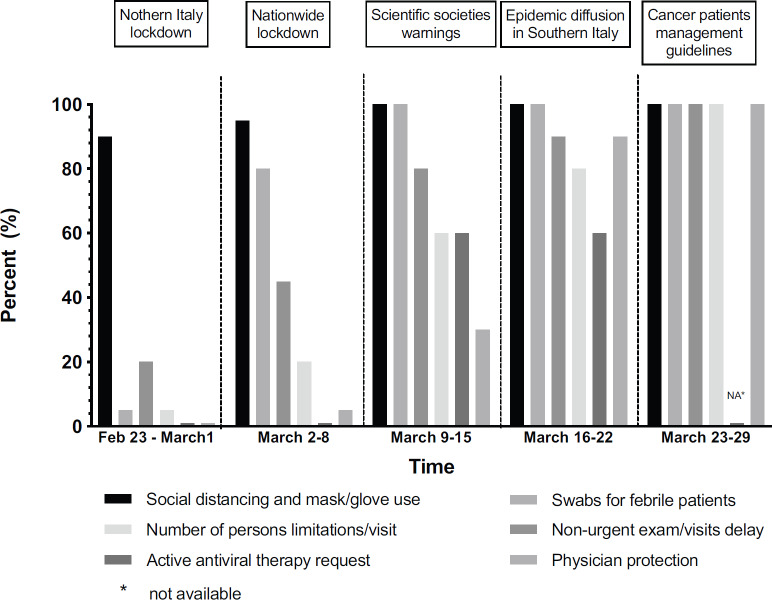
Actions taken in medical oncology units.

Figure 3 shows time-dependent actions—expressed as a relative percentage—taken in the medical oncology units included in the chat group. Actions put in place were also correlated time wise to government ordinances, such as partial and then global lockdown, and also guidelines release, epidemiological data and notification of infection spreading to health personnel. Chi-square test for trend was highly significant (*p* < 0.0001). The actions analysed were, at least in part, started before receiving detailed guidelines from the regulatory agencies dynamically coming from either regional or national government to manage the COVID-19 spread into the country. The first countermeasures have been represented by informing patients to adopt physical distancing, mask, and gloves use, which were mandatory for access to the hospitals. A second action was delaying non-urgent exams and follow-up visits, for instance, asymptomatic women with breast cancer under adjuvant hormonotherapy or males with prostatic carcinoma with a not increasing PSA. A third action has been the implementation of telephone or web triage for fever, coughs, dyspnoea and travel history of patients and their families, and possible contacts with other individuals tested positive for COVID-19, in case of risk, patients faced in quarantine in accord with their practitioners. Subsequently, only one person per visit has been allowed, and no caregiver allowed to stay on the wards (fourth action), and finally, in the last two weeks requirement for drugs potentially active against COVID-19, such tocilizumab, was preventively carried out [31].

**Figure 4.**
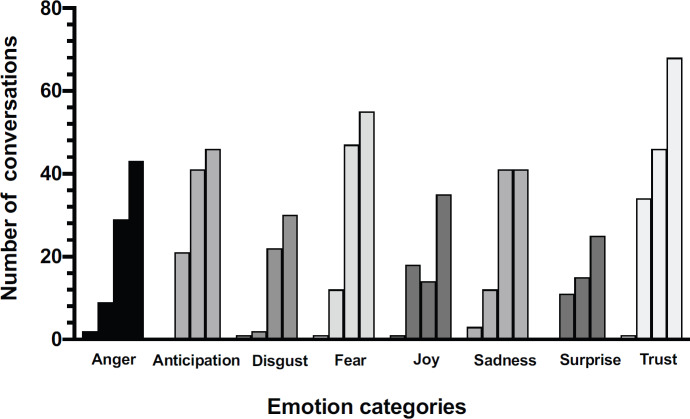
Weekly emotional trend according to crucial events.

**Figure 5.**
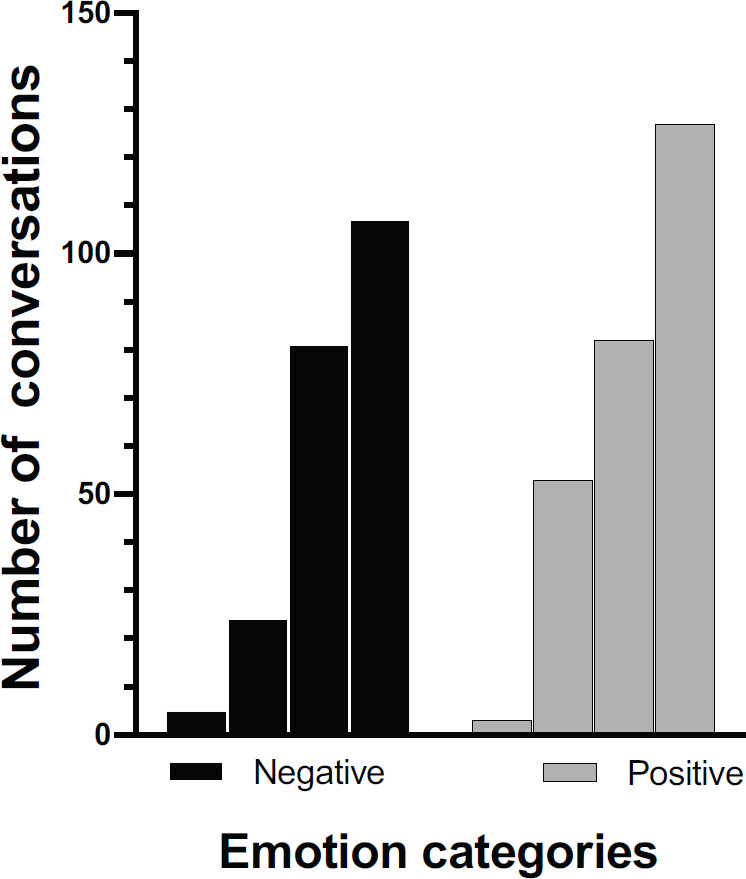
Weekly trend of positive versus negative reactions.

Figure 4 shows the distribution of emotions detected through the examination of the raw data and the results of sentiment analysis. The intensity of the scores related to the emotions evaluated is higher for the negative ones (Figure 5). The time-trend of the number of oncologists reporting positive emotions was slightly higher than those showing negative emotions. Fear, anger and sadness are dominant, while trust is represented by at least half of the dataset. A significant association between the different weeks and the pattern of emotions (positive or negative) was observed, Chi-square: 8.44, *p* = 0.38.

## Discussion

In the current COVID-19 epidemic scenario, the management of cancer patients and the modulation of the organisation of the Oncological Hospitals represent an urgent necessity worldwide [32]. Data concerning cancer patients during the COVID-19 pandemic are quite limited. In a series of 1,571 Chinese patients who required hospitalisation in intensive care units, invasive ventilation or had an ominous event, patients with cancer, especially when treated with chemotherapy, required urgent admission more frequently than non-neoplastic ones (39% versus 8%) [33]. In this rapidly changing setting, most warnings come from good common sense rather than based on objective controlled data.

To our knowledge, reports concerning the use of WM in any oncological setting are almost entirely occasional in the medical literature although it has been reported to be widely employed mainly because of doctors' perception of its advantages [14]. WM and other apps are used in endoscopic ultrasound fine needle aspiration (EUS-FNA) of pancreatic lesions, in self-monitoring melanoma, and chemotherapy monitoring [34–36]. Some of us reported the use of WM as a tool for the multidisciplinary management of patients with prostate cancer in daily practice [37]. A diagnostic and therapeutic agreement was reached in nearly 82% of cases, and these data were shared with patients. Doctors' satisfaction was measured by employing a questionnaire reporting an average satisfaction score of 7.8 on a 0–10 scale [37].

Due to its unplanned nature, this study lacks data concerning health-providers ethical issues and the importance of prioritising cancer patients according to their clinical characteristics and disease status. Although non-exhaustive, our report, however, indicates that among medical oncologists, the danger of the COVID-19 pandemic has been strongly perceived, and still is a crucial issue. Awareness of problems linked to the pandemic is demonstrated by the number and intensity of WhatsApp messenger-based group conversations between the medical directors belonging to the CIPOMO and the partly spontaneous, tentative countermeasures progressively taken in each institution even before health institutions promulgated specific guidelines and ordinances. Time trend analysis shows that after the first lag week since the beginning of the COVID-19 outbreak, the intensity of conversations between oncologists sharply increased in the two following weeks. A similar trend has occurred for actions taken and emotional reactions. The intensity of conversations was correlated to the volume of activity, being high-volume ones the most active ones.

This study also explored emotional expressiveness for eight specific types of emotions and identified those mainly expressed through the textual messages of medical oncologists examined in the sample. The intensity of the scores related to the emotions evaluated is slightly higher for the positive ones. These data indicate how strong the emotional impact is on medical oncologists. The results also show that oncologists’ concerns increased dramatically after first March with an increase in the intensity of the emotion expressed, such as fear, anger and sadness. The uncertainty of the imminent situation, although capable of causing cognitive dissonance and insecurity, seems to have stimulated determination to act as part of the Italian Health System. These considerations are inferred mostly from the evaluation of the trust emotion, which is continuously increasing during the weeks under analysis. Moreover, concerning the purely routine activity of medical care delivered to cancer patients and just outside of the high-intensity hospital wards reprogrammed to assist patients affected by COVID-19, oncologists have been challenged by the application of the selection criteria [29] for the normal-required oncological care. Their activation in the third week under review would explain in the sentiment analysis the leap in the intensity of emotion fear. This reaction may be the consequence of the feeling of discomfort of not making the right choice.

In a short time, the release of Government ordinances and extraordinary guidelines from scientific societies have profoundly changed the management of cancer patients, which are felt to be particularly at risk of life-threatening complications if infected with COVID-19 [32]. In this everchanging scenario of uncertainty, a large proportion of patients are currently managed via phone and web-based tools. WhatsApp messenger-based, although perfectible, is a useful tool to efficiently and rapidly share common thoughts, doubts and proactive ideas among medical personnel involved in the management of cancer patients.

## Limitations

The sample collected cannot be considered to reflect all topics of the conversations among oncologists involved. Moreover, the results of this analysis represent a snapshot of a particular period and have no evaluation comparison back and forth in time. In many unsupervised sentiment analysis applications, it is realistic to get an uncertain count of false positives and false negatives. This systematic prediction error can bias the true composition of sentiment in the data set.

## Conclusion

We can conclude that WhatsApp instant-messaging system is a useful and rapid tool to share information between healthcare professionals in the real world. This class of tools may allow coordinated actions which may ultimately result in an improvement of cancer patient management.

## Ethics approval and consent to participate

Not applicable.

## Consent for publication

Not applicable.

## Data availability

Not applicable.

## Conflicts of interest

The authors have no conflicts of interest to declare. Dario Piazza (project and data analyst manager), Alberto Firenze (epidemiologist), Michele Caruso, Massimiliano Spada, and Dario Giuffrida (medical oncologists), do not belong to the CIPOMO but they are allowed to participate. Vittorio Gebbia, as a professor of medical oncology, is, however, part of the mailing list and allowed to participate in CIPOMO-bases activities.

## Funding

An unrestricted grant from GSTU Foundation and the Risk Management Unit, Policlinic ‘P Giaccone’, University of Palermo, Italy, partially funded this work.

## Authors’ contributions

Data analysis and interpretation: VG, DP. Manuscript writing: all authors. Final approval of manuscript: all authors. Accountable for all aspects of the work: all authors.

## Figures and Tables

**Table 1.** Participating medical oncology institutions and messages statistics.

N. of institutions		17 (100%)
Type of institutions	Comprehensive cancer centres	5 (26%)
	General hospital	12 (63%)
	Academic	2 (11%)
Facilities/institution		
	Day hospital only	4 (22%)
	Day hospital + beds	15 (78%)
Patients burden		
	High volume	7 (27%)
	Low volume	12 (63%)
Total n. of conversations		956 (100%)
	Median/day	45 (range 2–58)
	Median/week	173 (range 12–386)
N. of conversations/unit		21.3
	Median/week	14 (range 0–147)
